# Supplementary material for: The interactome of intact mitochondria by cross-linking mass spectrometry provides evidence for coexisting respiratory supercomplexes
Source: Mol Cell Proteomics. 2017 Dec 8;17(2):216–32. doi: 10.1074/mcp.RA117.000470 (PMC5795388; doi:10.1074/mcp.RA117.000470)
Supplement: Supplemental Data [file supp_RA117.000470_133922_0_supp_23978_fzffwf.zip › spectra_annotation/mito_DR_spectra_annotation/432-1-16-1-16-1.pdf]

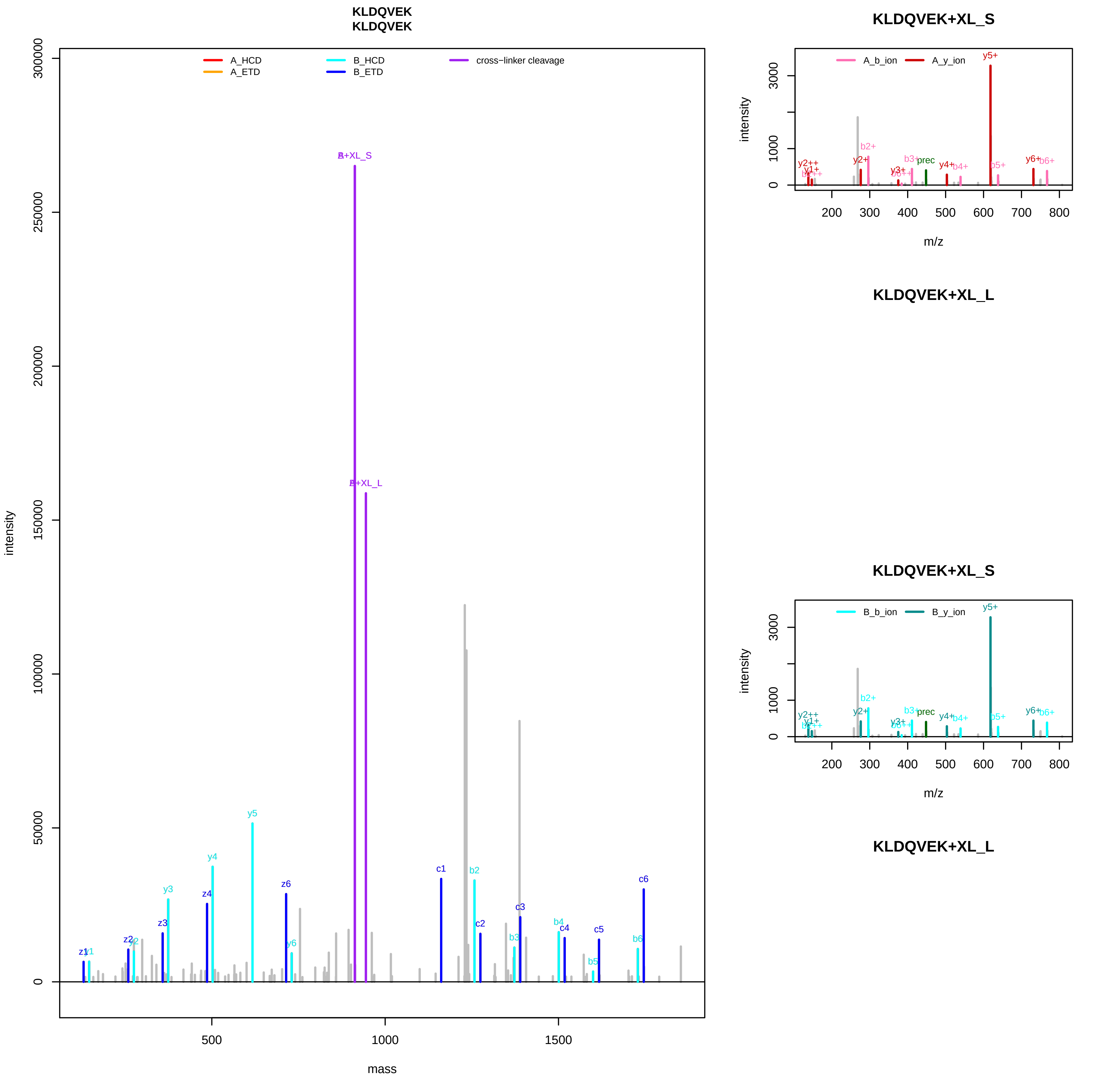

KLDQVEK
KLDQVEK
A_HCD
A_ETD
B_HCD
B_ETD
cross−linker cleavage
intensity
mass
0
50000
100000
150000
200000
250000
300000
500
1000
1500
z1
y1
z2
y2
z3
y3
z4
y4
y5
z6
y6
B+XL_S
B+XL_L
c1
b2
c2
b3
c3
b4
c4
b5
c5
b6
c6
KLDQVEK+XL_S
A_b_ion
A_y_ion
B_b_ion
B_y_ion
y5+
b2+
y2+
b3+
prec
y4+
b4+
b5+
y6+
b6+
y3+
y2++
y1+
m/z
KLDQVEK+XL_L
